# Upregulation of miR-21 in Cisplatin Resistant Ovarian Cancer via JNK-1/c-Jun Pathway

**DOI:** 10.1371/journal.pone.0097094

**Published:** 2014-05-27

**Authors:** Ileabett M. Echevarría-Vargas, Fatma Valiyeva, Pablo E. Vivas-Mejía

**Affiliations:** 1 Comprehensive Cancer Center, University of Puerto Rico, San Juan, Puerto Rico; 2 Department of Biochemistry, University of Puerto Rico, San Juan, Puerto Rico; University of Barcelona, Spain

## Abstract

Cisplatin has been the most accepted drug for the treatment of ovarian cancer for almost 40 years. Although the majority of patients with ovarian cancer respond to front-line platinum combination chemotherapy, many patients will develop cisplatin-resistance disease, which is extremely rapid and fatal. Although various mechanisms of cisplatin resistance have been postulated, the key molecules involved in such resistance have not been identified. MiRNAs are endogenously expressed small non-coding RNAs, which are evolutionarily conserved and function as post-transcriptional regulators of gene expression. Dysregulation of miRNAs have been associated with cancer initiation, progression and drug resistance. The oncogenic miRNA-21, one of the best-studied miRNAs, is upregulated in almost all human cancers. However, the regulation of miR-21 in cisplatin resistant ovarian cancer cells has not been assessed. In this study, we measured the miR-21 expression by real-time PCR and found upregulation of miR-21 in cisplatin resistant compared with cisplatin sensitive ovarian cancer cells. Chromatin immunoprecipitation studies demonstrated the association of the c-Jun transcription factor to the pri-mir-21 DNA promoter regions. Blocking the JNK-1, the major activator of c-Jun phosphorylation, reduced the expression of pre-mir-21 and increased the expression of its well-known target gene, PDCD4. Overexpression of miR-21 in cisplatin sensitive cells decreased PDCD4 levels and increased cell proliferation. Finally, targeting miR-21 reduced cell growth, proliferation and invasion of cisplatin resistant ovarian cancer cells. These results suggest that the JNK-1/c-Jun/miR-21 pathway contributes to the cisplatin resistance of ovarian cancer cells and demonstrated that miR-21 is a plausible target to overcome cisplatin resistance.

## Introduction

Ovarian cancer (OvCa) accounts for near 3% of all cancers in the Western world [1]. Typical treatment for women with OvCa is cytoreduction followed by chemotherapy [2]. Despite the high initial response rate to cisplatin-based compounds as the first-line chemotherapy, most ovarian carcinomas relapse [2],[3]. Postulated mechanisms of cisplatin resistance include decreased intracellular accumulation of cisplatin, increased intracellular levels of certain sulfur-containing macromolecules, and increased DNA repair [4]. Evidence suggests that inactivation of intrinsic cell death pathways, activation of cell survival pathways, and dysregulation of oncogenes, tumor suppressor genes, and microRNAs, contribute to the cisplatin resistance of ovarian cancer cells [3],[5],[6]. However, the exact mechanism by which ovarian cancer cells become resistant to cisplatin treatment is presently unknown.

MicroRNAs (miRNAs) are naturally occurring small (21–22 base pairs) non-coding RNAs, which recognizes mainly the 3′-UTR region of messenger RNA (mRNA) and inhibit protein synthesis [7]. Recent reports indicate that dysregulation of miRNAs, and their target genes, promote cancer initiation, progression and drug resistance [6],[8],[9]. For these reasons, miRNAs have been proposed as a diagnostic, prognostic and targets for cancer therapy [10]. One of the best studies miRNAs is miR-21 which is overexpressed in most cancers and displays oncogenic activity [11]. In HER2(+) breast cancers overexpression of miR-21 conferred resistance to trastuzumab therapy [12]. Nam et al., found miR-21, miR-203, and miR-205 as overexpressed in serous ovarian carcinoma versus normal ovarian tissue; miR-21 being present in 85% samples [13]. Xie et al. observed that in the A2780 human ovarian cancer cells, miR-21 modulates the hypoxia-inducible factor-1α (HIF-1α), and the resistance of paclitaxel [14]. MiR-21 exerts its biological role by targeting key genes controlling cell growth and proliferation. However, in solid tumors including ovarian cancer, the tumor suppressor gene programmed cell death 4 (PDCD4) appears to be one of the most important functional miR-21 targets [15],[16],[17]. The central role of PDCD4 in ovarian cancer is further supported by observations that lower levels of this tumor suppressor correlate directly with poor prognosis of ovarian cancer patients [15]. More recently, Chan et al. showed that miR-21 is overexpressed in cisplatin sensitive compared with cisplatin resistant cells, and that miR-21 inhibition induced apoptosis and increases PDCD4 levels [18].

The upstream molecular events that accounts for the miR-21 overexpression in ovarian cancer cells need further study. Reports indicate that the pri-mir-21 DNA promoter regions contain recognition elements for c-Jun and STAT3 transcription factors [19]. In colon cancer cells, curcumin reduced miR-21 via AP-1 (transcription factor complex composed of c-Jun and c-Fos, mainly) regulation, and in the human promyelocytic cell line, HL-60, phorbol 12-myristate 13-acetate (PMA) induced miR-21 expression by AP-1 activation [17],[20]. MiR-21 upregulation by AP-1 was also observed in certain chemoresistant side populations of cancer stem cells [21].

Because the recognized role of miR-21 in cancer initiation, progression and drug resistance, we investigated the regulation of miR-21 in cisplatin resistant ovarian cancer cells. We used real-time polymerase chain reaction (PCR) and found that cisplatin resistant ovarian cancer cells express higher miR-21 levels compared with cisplatin sensitive cells. We then analyzed the possible mechanism of miR-21 upregulation. Treatment of cells with SP600125, an inhibitor of the c-Jun phosphorylation, reduced pre-mir-21 expression in cisplatin resistant ovarian cancer cells. Chromatin immunoprecipitation (ChIP) studies showed higher c-Jun phosphorylation (p-c-Jun) protein levels bound to the pri-mir-21 DNA promoter region in cisplatin resistant compared with cisplatin sensitive ovarian cancer cells. Overexpression of miR-21 in cisplatin sensitive cells, increased cell proliferation, and decreased the PDCD4 protein levels. Opposite, miR-21 oligonucleotide inhibitor (antagomir), significant inhibited cell growth, proliferation and the invasion ability of A2780CP20 cells. This data demonstrate that upregulation of the JNK-1/c-Jun pathway leads to the aberrant increasing of miR-21, and propose to miR-21 as a potential target to overcome cisplatin resistance of ovarian cancer cells.

## Materials and Methods

### Chemicals and reagents

Cisplatin was purchased from Sigma-Aldrich (St. Louis, MO) and reconstituted in 0.9% NaCl. SP600125 (Sigma-Aldrich, St. Louis, MO) was reconstituted in 100% DMSO up to final concentration of 10 mM. When added to the cells, the final concentration of DMSO on the culture medium was less than 0.1%.

### Cells and culture conditions

The human ovarian epithelial cancer cells SKOV3ip1, HEYA8, and A2780CP20 cells have been described elsewhere [22],[23],[24],[25],[26]. A2780 and A2780CIS cells were purchased from the European Collection of Cell Cultures (ECACC). The cells were propagated *in vitro* in RPMI-1640 medium (Thermo Scientific, Logan, UT, USA) supplemented with 10% fetal bovine serum (FBS) (Thermo Scientific) and 0.1% antibiotic/antimycotic solution (Thermo Scientific) and maintained at 37°C in 5%CO_2_/95% air. All tumor cell lines were screened using Mycoplasm removal agent as described by the manufacturer (AbD Sertotec, NC, USA). *In vitro* assays were performed at 70–85% cell density. A2780CP20 cells (5×10^5^ cells/dish) were treated with 10 µmol/L of SP600125 for eight hours. Cisplatin concentrations inhibiting 50% of cell growth (IC_50_) were calculated after 72-hr drug incubation (cisplatin doses: 100, 10, 1, 0.1, and 0.01 µM, final concentrations). Cell growth was measured with the Alamar blue dye as previously described [27].

### Microarray analysis of gene expression

Total RNA was isolated from A2780 and A2780CP20 ovarian cancer cell lines with the GenElute Mammalian Total RNA Purification Kit (Sigma-Aldrich). The RNA concentration was read in a nanodrop. RNA quality was verified in a Bioanalyzer (Agilent Technologies, Inc., Santa Clara, CA). One hundred nanograms of total RNA were used to cDNA synthesis. Complementary cDNA was synthesized, labeled, fragmented and hybridized to the Affymetrix GeneChip Gene 1.0 ST Human Array. After 16 hours of incubation at 45°C, the arrays were washed, stained and scanned (Affymetrix Model 3000 scanner), and data was analyzed using the Partek Genomics Suited (Partek, St. Louis, MO). Experiments were performed in duplicate. Two-way ANOVA was used to determine differences in gene expression (p<0.05 fold change 2). 1359 probes (4%) sets showed ±2-fold change between A2780CP20 and A2780 cells. Additional filtering (±3-fold change, and p<0.003) showed 321 probes (0.96%) changed between A2780CP20 and A2780 cells.

### RNA isolation and cDNA synthesis

Total RNA (including miRNAs) was isolated using the *mir*Vana miRNA isolation kit from Ambion (Life Technology, Grand Island, NY). RNA was converted into cDNA with the Enhanced Avian RT first strand synthesis kit from Sigma-Aldrich. In brief, total RNA (1 µg), 500 µM dNTP and 3.5 µM oligo(dT)_23_ were mixed and water was added up to 10 µL total volume. Samples were mixed, centrifuged and heated at 70°C for 10 min. This mixture was combined with 1 µL enhanced avian RT, 2 µL 10X buffer, 1 µL RNase inhibitor, and water up to 20 µL final volume. Samples were incubated at 25°C for 15 min following by 45°C for 50 minutes. cDNA synthesis to detect mature miRNAs was performed with the All-in-One miRNA qRT-PCR detection kit (GeneCopoeia, Rockville, MD). In brief, cDNA synthesis mixture contained 1 µg of total RNA, 1 µL Poly(A) Polymerase, 1 µL RTase Mix, 5 µL 5X PAP/RT Buffer and water up to 25 µL final volume. The revere transcription reaction was incubated at 37°C for 60 min, and 85°C for 5 min in a Veriti thermal cycler (Applied Biosystems, Carlsbad, CA).

### Polymerase Chain Reaction (PCR) and SYBR-I-based real-time PCR

PCR was performed in a Verity 96 wells thermal cycler (Applied Biosystems). In brief, 12.5 µL JumpStart REDTaq ReadyMix 1X (Sigma), 0.5 µL forward, and 0.5 µL reverse primers (0.4 µM final concentration of each), 2 µL of the cDNA product, and water up to 50 µL final volume. The cycling conditions were one cycle at 94°C for 5 min, followed by 30 cycles at 95°C for 15 sec (denaturation), 60°C for 30 sec (annealing), and 72°C for 30 sec (extension). A final extension step was performed at 72°C for 10 min. PCR products were separated by electrophoresis in 1% agarose gel. Images were obtained (after gel staining with ethidium bromide) in a FluorChem 8900 (Alpha Innotech). SYBR-I-real time PCR to assess the mature miR-21 levels were performed with the All-in-One miRNA qRT-PCR detection kit (GeneCopoeia) in a StepOne plus real-time PCR thermal cycle system (Applied Biosystems). The PCR reactions mix contained 10 µl 2X All-in-One qPCR Mix, 2 µl All-in-One miRNA qPCR primer, 2 µL universal adaptor PCR primer, 2 µL first strand cDNA, and 4 µL of water. Specific primers for miR-21 and U6 (internal standard) were used (GeneCopoeia). Cycling conditions: one cycle of 15 min at 95°C, and 40 cycles of 15 sec at 94°C, 30 sec at 60°C and 30 sec at 72°C [28]. Melt curve analysis was always performed at the end of the PCR reaction. Relative miR-21 expression was calculated with the ΔΔCt-method [29],[30],[31].

### Western blot analysis

Cells were collected and washed twice with Phosphate Buffer Saline (PBS), harvested and stored at −80°C until processed. For cytosolic and nuclear extraction, cells were resuspended in cytoplasmic buffer (0.5% Nonidet P-40, 10 mM KCl, 10 mM Hepes, pH 7.9, and 1X protease inhibitor) for 15 min on ice and centrifuged at 14,000 rpm at 4°C [32]. The supernatants (containing the cytoplasmic fraction) were collected. The remaining pellets were washed twice with the cytoplasmic buffer following by the addition of nuclear buffer (400 mM NaCl, 20 mM Hepes, pH 7.9 and 1X protease inhibitor). Samples were incubated for 15 min on ice, centrifuged for 10 min at 4°C and supernatant (containing the nuclear protein fractions) were collected. For total proteins extraction, cells were lysed with lysis buffer (150 mM NaCl, 1% Triton-X, 0.4 mM NaF, 0.4 mM NaVO_4_, 25 mM Tris-HCl, pH 7.6 and 1X protease inhibitor) for 45 min on ice, centrifuged for 10 min at 4°C and the supernatants were collected. Protein concentration was determined using Bio-Rad Protein Reagents (Bio-Rad, Hercules, CA). Protein lysates (50 µg) were separated by SDS-PAGE, blotted onto membranes, and probed with the appropriate dilution of each primary antibody. Membranes were rinsed and incubated with the appropriate horseradish peroxidase-conjugated secondary antibody, rinsed again, and the bound antibodies were detected using enhanced chemiluminescence (GE Healthcare, Piscataway, NJ) following by autoradiography in a FluorChem 8900 (Alpha Innotech Corporation, San Leandro, CA). Primary anti-bodies: anti-phospho-SAPK/JNK (Thr183/Tyr185), anti-SAPK/JNK, anti-phospho-c-Jun (Ser73), anti-c-Jun, anti-phospho-ERK (Thr202/Tyr204), anti-ERK kinase, anti-STAT3, anti-histone H3 (Cell Signaling, Danvers, MA), anti-PDCD4 (Rockland, Gilbertsville, PA) and anti-β-actin (Sigma-Aldrich). Secondary antibodies used were anti mouse and anti rabbit IgG horseradish peroxidase (HRP)-linked, anti rabbit (Cell Signaling).

### Chromatin immunoprecipitation (ChIP)

A2780CP20 and A2780 cells were collected and crosslinked with 1% formaldehyde for 10 min at 37°C. Cells were lysed in sodium dodecyl sulfate (SDS) lysis buffer (1% SDS, 10 mM EDTA, 50 mM Tris, pH 8.1 and protease and phosphatase inhibitors). Samples were sonicated (4°C) using a Branson 250 sonicator with 10 continued pulses of 10 seconds (sec) each. The sonicated lysates were pre-cleared with protein A or G magnet beads overnight at 4°C. Lysates were immunoprecipitated with 5 or 10 µg of antibody/magnet beads against p-c-Jun, Pol-II (Santa Cruz), c-Jun (BD Transduction laboratory, Franklin Lakes, New Jersey, USA) or IgG (Abcam, Cambridge, MA, USA) overnight at 4°C. Samples were washed five times for 5 min each at 4°C with wash buffer (RIPA) (50 mM Hepes-KOH pH 7.6, 500 mM LiCl, 1% NP-40, 0.7% deoxycholic acid, 1 mM EDTA, and 1X protease and 1X phosphatase inhibitors). Samples were washed once with 1X TE buffer and eluted with SDS elution buffer (1% SDS, 10 mM EDTA, 50 mM Tris pH 8.1, and 1X protease and 1X phosphatase inhibitors). Following elution, samples were reverse cross-linked at 65°C for 4 hours, treated with RNase A (0.2 µg/ml) at 37°C for 2 hours, mixed with CaCl_2_ (300 mM CaCl_2_ in 10 mM Tris pH 8.0) and Proteinase K (0.2 mg/ml) at 37°C overnight. The immunoprecipitated DNA was isolated using QiAquick PCR Purification Kit (Qiagen). DNA was quantified by SYBR-1-based real-time PCR using a StepOne Plus Thermocycler (Applied Biosystems). In brief, PCR reaction mix contained 10 µL SYBR-green, 0.5 µL forward primer, 0.5 µL reverse primer (0.25 nM final concentration), 2 µL of DNA sample and 7 µL of nuclease free water. Cycling conditions: one cycle of 10 min at 95°C, and 40 cycles of 30 sec at 95°C, 30 sec at 60°C and 30 sec at 72°C.

### Transient and stable transfections

Ectopic miR-21 expression was performed in A2780 cells. Briefly, A2780 (2×10^4^ cell/ml) cells were seeded in six well plates. For each well, 1 µg of pCMV-MIR21 or 1 µg of empty vector (pCMV-EV) (both from OriGene Technologies, Inc. Rockville, MD) and 1 µL of MegaTran 1.0 transfection reagent (OriGen) were diluted in 98 µL of Opti-MEM I (Life Technologies). pCMV vector contains a green fluorescein protein (GFP) cassette. The mixture was incubated for 10 min at room temperature and added to the cells. Twenty-four hours later, the medium was replaced with fresh RPMI-1640 (10% FBS, 0.1% antibiotic/antimycotic solution and 500 µg/mL G418 disulfate salt solution). After 2–3 weeks, independent colonies were picked and cultured separately as independent clones. The transfection efficiency was monitored by immunofluorescence as follows: A2780 clones were seeded in microscope slides overnight. Then, cells were fixed with 4% paraformaldehyde, blocked with 0.3% H_2_O_2_ in methanol for 10 min and 10% FBS for 20 min. Cells were incubated with anti-GFP antibody (dilution 1∶250) or DAPI (dilution 1∶5000 from Santa Cruz Biotechnology Inc., Dallas, Texas, USA) overnight. The next day, slides were washed, and the secondary antibody anti-rabbit (dilution 1∶5000) was added. Cover slips were placed on the microscope slides and fixed with Aqueous-mounting medium (Abcam). Cells were visualized and photographed in an Olympus 1X71 inverted microscope (Center Valley, PA). To inhibit miR-21, we transiently transfect A2780CP20 cells with a miR-21 oligonucleotide (Life Technology, Grand Island, NY, USA) and a negative oligonucleotide inhibitor as a control (Life Technology). MiRNA inhibitors were always mixed with HiPerfect transfection reagent (Qiagen, Valencia, CA, USA), and Opti-MEM I growth medium (Life Technologies).

### Small interference RNA (siRNA) transfection

To silence human c-Jun (NM_002228) two siRNAs targeting the sequences: 5′-CCTTCTATGACGATGCCCT-3′, and 5′-GATGGAAACGACCTTCTAT-3′ were used (Sigma-Aldrich). A non-silencing siRNA (NC-siRNA) was used as the negative control (Sigma-Aldrich). In brief, A2780CP20 (2.25×10^5^ cells) were seeded in petri dishes. Twenty-four hours later, 200 nM of siRNA (final concentration) were mixed with HiPerfect transfection reagent (Qiagen) at 1∶2 ratio (siRNA:transfection reagent) in Opti-MEM I growth medium. The mix was incubated for 15 minutes at room temperature and added to the cells. Cells were collected after 24 hours and store at -80°C until use.

### Cell growth and proliferation

A2780CP20 (2×10^4^ cell/ml) were seeded in six well plates. Twenty-four hours later a transfection reaction mix containing miR-21 inhibitor (Life Technology) (50 nM final concentration) and HiPerfect transfection reagent (Qiagen) in a 1∶4 ratio, was added to the cells. Six hours later, the cultured medium was removed and replaced by RPMI-1640 medium (10% FBS). To assess cell viability, seventy-two hours after transfection, live cells were collected and counted in the presence of 0.5% Trypan blue using a Countess automated cell counter (Invitrogen, Grand Island, NY). Cell proliferation was assessed with a colony formation assay. Briefly, eight hours after transfection, 1000 cells were seeded in 10 cm-Petri dishes. Ten days later, colonies were stained with 0.5% crystal violet in methanol, and counted using the microscope Nikon eclipse TS100.

### In vitro invasion assay

Cells (2.5×10^4^ cells/ml) were plated in a petri dish. Twenty-four hours later a transfection reaction mix containing miR-21 inhibitor (Life Technology) (50 nM final concentration) and HiPerfect transfection reagent (Qiagen) in a 1∶2 ratio, was added to the cells. The next day, 600 µL of diluted matrigel (serum-free RPMI medium) was putted into upper chamber of 6-well Transwell plates (Corning Incorporated, Lowell, MA). The chamber was incubated at 37°C at least 4 to 5 hours for gelling. MiR-21 inhibitor-transfected cells were collected and resuspended in serum-free RPMI-1640 medium at a density of 1.5×10^5^ cells/ml. The matrigel was gently washed with warmed serum free-RPMI-1640 medium, and 1.0 mL of the cell suspension was putted onto the matrigel. The lower chamber of the transwell was filled with 1.0 mL RPMI-1640 medium (10% FBS), and the plate was incubated at 37°C for 48- hr. The transwells were removed from the 6-well plates and stained with the PROTOCOL HEMA 3 Stain set (Fisher Scientific Company, Kalamazoo, MI). The non-invaded cells were scraped off on the top of the transwell with a cotton swab. The number of invaded cells was counted in four microscope fields (10X) per condition. Percentage of invasion was calculated relative to the number of invaded cells in the control miRNA inhibitor well, which was taken as 100%.

### Statistical analysis

Student's t-test was performed using GraphPad Prism version 5.04 for Windows, GraphPad Software, San Diego California USA, www.graphpad.com. All experiments were performed in triplicate and p-value <0.05 for a two-sided test was considered statistically significant.

## Results

### Expression of miR-21 and miR-21-related molecules in ovarian cancer cells

**Figure 1:**
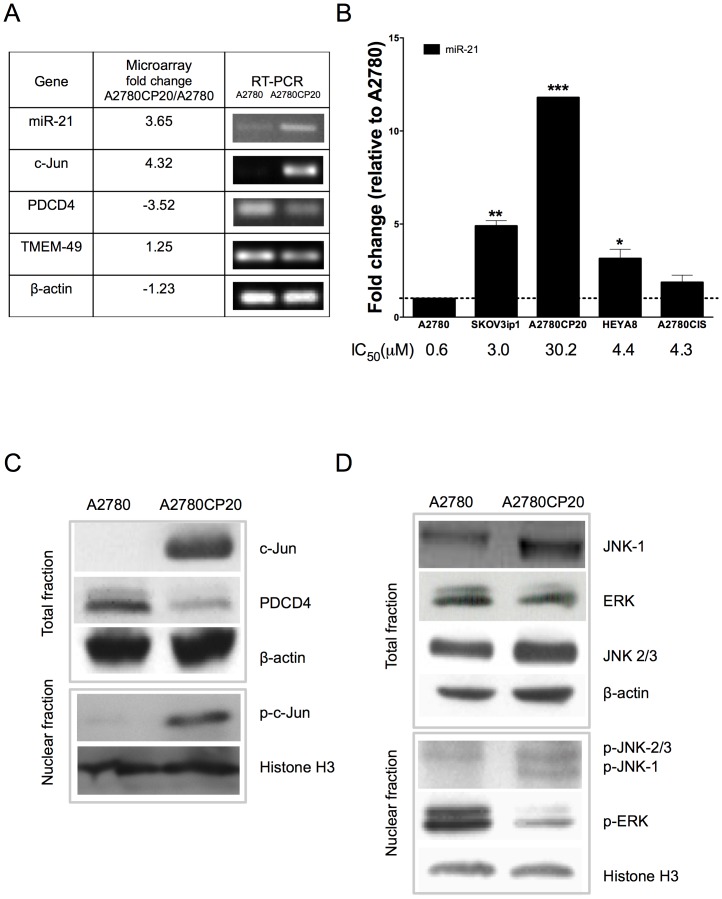
RT-PCR and western blot analysis of miRNA-21-related molecules. (**A**) Validation of microarrays by RT-PCR. (**B**) MiR-21 levels in a panel of ovarian cancer cells. MiR-21 levels were expressed relative to the A2780 cells miR-21 levels. IC50s were calculated after 72-hr treatment of cells with different concentrations of cisplatin as described in the “Material and Methods” section. (**C**) Evaluation of c-Jun and p-c-Jun protein expression in A2780 and A2780CP20 cells. (**D**) Protein expression analysis of MAPKs in total and nuclear fractions of A2780 and A2780CP20 cells. Expression level in Figures A, C and D are without cisplatin treatment. *p<0.05, **p<0.01, ***p<0.001 compared to control. Columns represent the means of triplicates ± S.E.M.

In a preliminary microarray analysis performed in our laboratory, we identified 320 genes differentially expressed (fold change >3 and p-value < 3×10^−4^) in A2780CP20 (cisplatin resistant) *vs.* A2780 (cisplatin sensitive) cells (Table S1). The microarray data was deposited in the Gene Expression Omnibus (GEO): accession: http://www.ncbi.nlm.nih.gov/geo/query/acc.cgi?acc=GSE51683. Whereas miR-21 and c-Jun were upregulated, PDCD4 was downregulated in A2780CP20 compared with A2780 cells (Table S1). RT-PCR experiments corroborated the microarray data (Figure 1A). MiR-21 gene is located in the intron 10 of the TMEM-49 gene [17]. The messenger RNA (mRNA) levels of TMEM-49 and β-actin were not different in A2780CP20 and A2780 cells (Figure 1A), which corroborate previous finding that pre-mir-21 and TMEM-49 are independently regulated [33]. Figure 1B shows that the miR-21 levels correlate with the sensitivity of ovarian cancer cells to cisplatin treatment (IC_50_, Figure 1B). Western blot analysis (Figure 1C) showed that the total c-Jun and p-c-Jun protein levels were higher in A2780CP20 compared with A2780 cells. However, the PDCD4 protein levels showed opposite results (Figure 1C). In other cell lines, the Signal Transducer and Activator of Transcription 3 (STAT3) regulate miR-21 expression [17]. However, the STAT3 protein levels were similar in A2780CP20 and A2780 cells (Figure S1), which rule out the possibility that STAT3 account for the higher miR-21 levels in A2780CP20 compared with A2780 cells. These results suggest that c-Jun is responsible for miR-21 expression levels in cisplatin resistant ovarian cancer cells.

It is well known that the major activator of c-Jun is JNK-1 [34]. Thus, we assessed the protein levels of JNK in A2780 and A27870CP20 cells. The total JNK-1 and nuclear p-JNK-1protein levels were higher in A2780CP20 compared with A2780 cells. The protein levels of p-JNK-2/3 and total JNK-2/3, the two other JNK-1 isoforms, were similar in both cells lines (Figure 1D), which suggest that the JNK-1/c-Jun cascade regulate miR-21 in A2780CP20 cells. Evidence indicates that the extracellular signal regulated kinase, ERK can activate c-Jun [35],[36]. Although the total ERK protein levels were similar in both cells lines, the p-ERK protein levels were lower in A2780CP20 compared with A2780 cells ruled out the possibility that activated ERK account for the higher miR-21 levels in A2780CP20 compared with A2780 cells.

### Effect of JNK-1/c-Jun inhibition in miR-21 and PDCD4 expression

**Figure 2:**
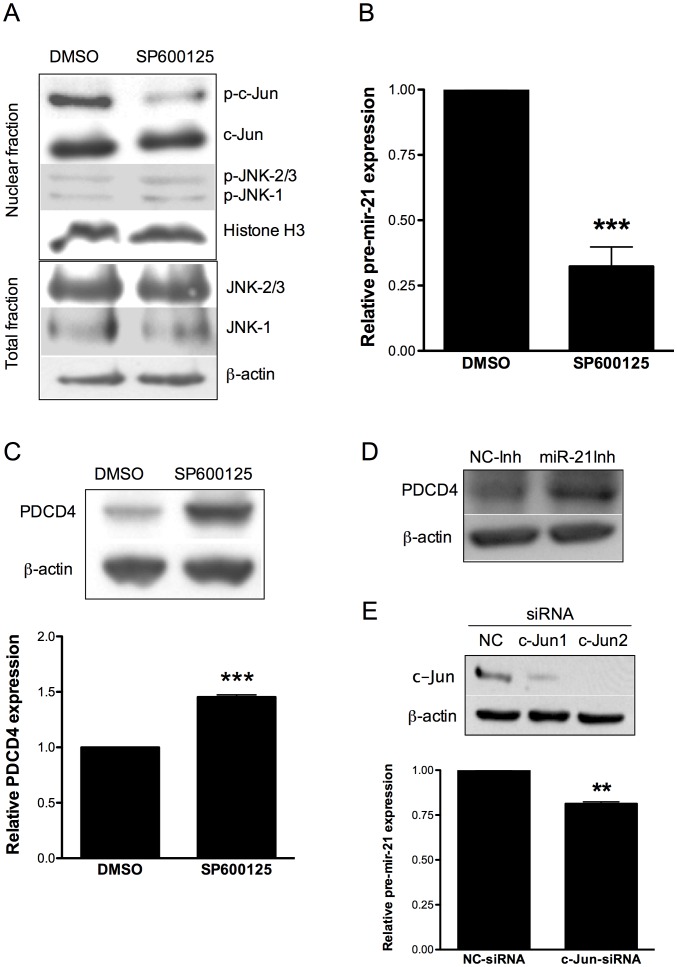
Effect of JNK-1 inhibition in miR-21 and PDCD4 expression. A2780CP20 cells were treated with 10 µM SP600125. (**A**) Western blot shown the inhibition of p-c-Jun after treatment with SP600125 in A2780CP20 cells compared to control (DMSO). (**B**) SYBR-I-based real-time PCR was performed to calculate the relative pre-mir-21 expression in A2780CP20 cells after treatment with SP600125 inhibitor. (**C**) Western blot and densitometric analysis of PDCD4 protein expression levels after treatment of A2780CP20 cells with SP600125. (**D**) PDCD4 protein expression levels after transfection of A2780CP20 with miR-21 oligonucleotide inhibitor. (**E**) A2780 CP20 cells were transiently transfected with two c-Jun-targeted siRNAs as described in the “Materials and Methods” section. Western blot analysis shows that both c-Jun-siRNAs decreased the c-Jun levels. SYBR-I-based real-time PCR was performed (see “Materials and Methods” section) to calculate the relative pre-mir-21 expression levels in A2780CP20 cells after siRNA-mediated c-Jun silencing. *p<0.05, **p<0.01, ***p<0.001 compared to control. Columns represent the means of triplicates ± S.E.M.

To further determine whether JNK-1 regulates c-Jun and miR-21 expression in cisplatin resistant cells, we treated A2780CP20 cells with SP600125, a well-known chemical inhibitor of c-Jun phosphorylation by JNK-1 [34]. Incubation of A2780CP20 cells with SP600125 decreased the p-c-Jun levels, as expected (Figure 2A). No significant changes in the protein levels of c-Jun, total and p-JNK-1 or total and p-JNK-2/3 were observed following treatment of A2780CP20 cells with SP600125 inhibitor (Figure 2A). Real-time PCR showed a significant reduction (60% and *p<0.05) in the pre-mir-21 levels following treatment of A2780CP20 cells with SP600125 (Figure 2B). The same inhibitor caused an increase in the PDCD4 protein levels as observed in the western blot of Figure 2C. Furthermore, inhibition of miR-21 with a miR-21 specific oligonucleotide inhibitor (antagomiR) increased the PDCD4 protein levels (Figure 2D), which confirmed previous results that miR-21 regulates PDCD4 in ovarian cancer cells [37],[18]. Furthermore, transient transfection of A2780CP20 cells with a siRNA against c-Jun significantly (**p<0.01) inhibited pre-miR-21 expression (Figure 2E). Western blot analysis showed that treatment of the cisplatin sensitive A2780 cells with SP600125 did not affect the levels of p-JNK-1, JNK-1, p-JNK-2/3, JNK2/3, c-Jun, pre-miR-21 or PDCD4 (Figure S2). These results suggest that JNK-1/c-Jun pathway lead to miR-21 overexpression in cisplatin resistant ovarian cancer cells.

### Analysis of the c-Jun binding to miR-21 DNA promoter regions

**Figure 3:**
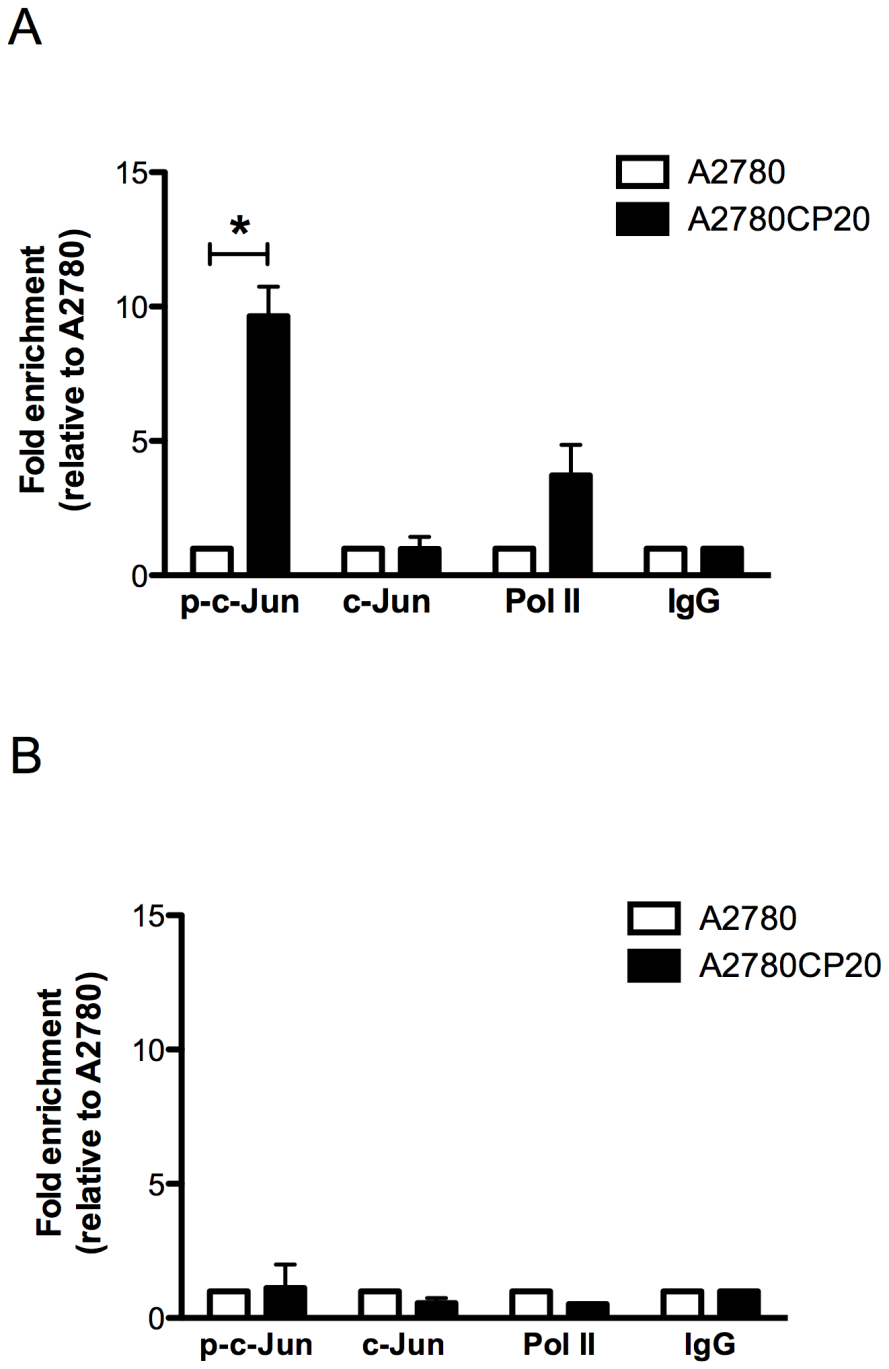
Chromatin immunoprecipitation assay (ChIP). ChIP assay was performed as described in the “Materials and Methods” section. (**A**) SYBR-I-based real-time PCR amplification of the region containing the c-Jun recognition sequence in the pri-miR-21 DNA. The phospho-c-Jun levels bound to the pri-miR-21 promoter was higher in A2780CP20 cells compared with A2780 cells. (**B**) SYBR-I-based real-time PCR amplification of a DNA region far of the pre-mir-21 promoter was performed as a control. *p<0.05 compared to control. Columns represent the means of triplicates ± S.E.M.

To definitely demonstrate that c-Jun is responsible of the higher miR-21 levels in A2780CP20 compared with A2780 cells, we assessed the pri-mir-21 promoter DNA regions associated with p-c-Jun by ChIP analysis. The amount of DNA immunoprecipitated with p-c-Jun antibody in both cell lines was amplified with a pair of primers encompassed the c-Jun recognition elements in the pri-mir-21 promoter region (Figure S3 and Table S2). Other pair of primers amplifying a DNA region far from the c-Jun recognition sites was used as a control (Table S2). More than five times of DNA levels were pulling out in A2780CP20 than in A2780 cells after immunoprecipitation with the p-c-Jun antibody (Figure 3A). No significant differences in DNA amounts were observed after immunoprecipitation with c-Jun, Pol- II or IgG antibodies (Figure 3A) which demonstrated that p-c-Jun binds specifically to pri-mir-21 promoter regions, and that more p-c-Jun protein levels are higher in A2780CP20 than in A2780 cells. No significant differences in the DNA levels were observed when the PCR was performed with the non-promoter DNA primers after immunoprecipitation with the p-c-Jun antibody (Figure 3B).

### Effect of miR-21 overexpression in the sensitivity of ovarian cancer cells to cisplatin treatment

**Figure 4:**
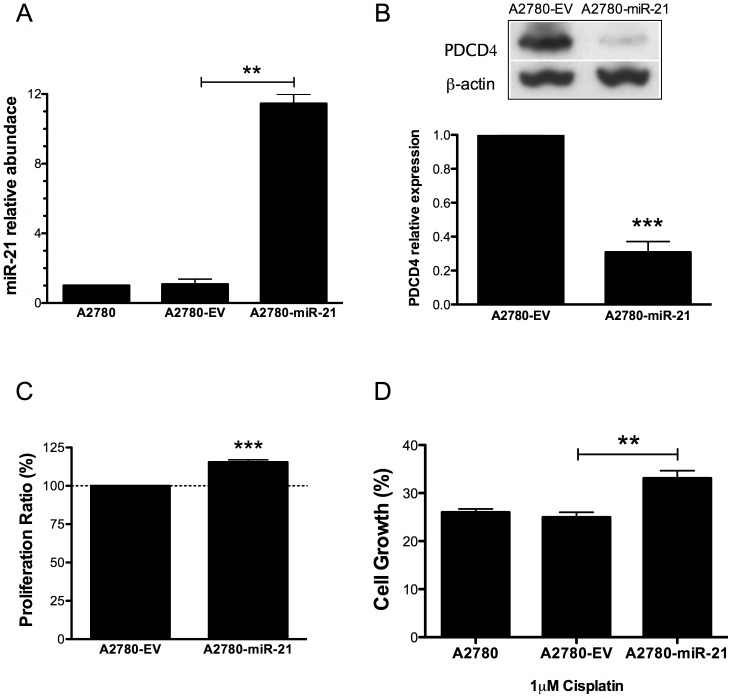
Effect of pre-mir-21 overexpression in A2780 cells. (**A**) A2780 cells were stably transfected with pCMV-miR21 or empty pCMV-EV vectors. The miR-21 expression was quantified by qRT-PCR. (**B**) Western blot analysis shows a decreased expression of PDCD4 levels in A2780-miR-21 compared with A2780-EV cells. (**C**) Overexpression of miR21 increased cell proliferation (13%, ***p<0.001) in A2780-miR-21 compared with A2780-EV cells. (**D**) A2780-miR-21 overexpressed clones were more resistant to cisplatin-induced cell death compared with untransfected A2780 cells or with the A2780-EV cells. **p<0.01. Columns represent the means of triplicates ± S.E.M.

To test whether miR-21 contributes to the cisplatin resistance of ovarian cancer cells, we ectopically expressed pre-mir-21 in A2780 cells (Figure S4 and S5). Figure 4A, shows that compared with untransfected A2780 cells or with the empty vector (clone 1, Figure S4), stable transfection of pre-mir-21 (clone 1, Figure S4) increased the miR-21 mature levels. The PDCD4 protein levels were also significantly decreased (69%, ***p<0.001) in the pre-mir-21 A2780 clone (A2780-miR-21) compared with the empty vector clone (A2780-EV) (Figure 4B). Ectopic expression of pre-mir-21 in A2780 cells resulted in a significant increase in cell proliferation (15.4%, ***p<0.001) compared with A2780-EV cells (Figure 4C). In addition, cisplatin treatment (1 µM) of A2780-miR-21 cells resulted in significant (approximately 15%, **p<0.01) reduction of cell growth inhibition compared the cisplatin treatment of A2780-EV cells (Figure 4D).

### Effect of miR-21 inhibition in cell growth, proliferation and invasion

**Figure 5:**
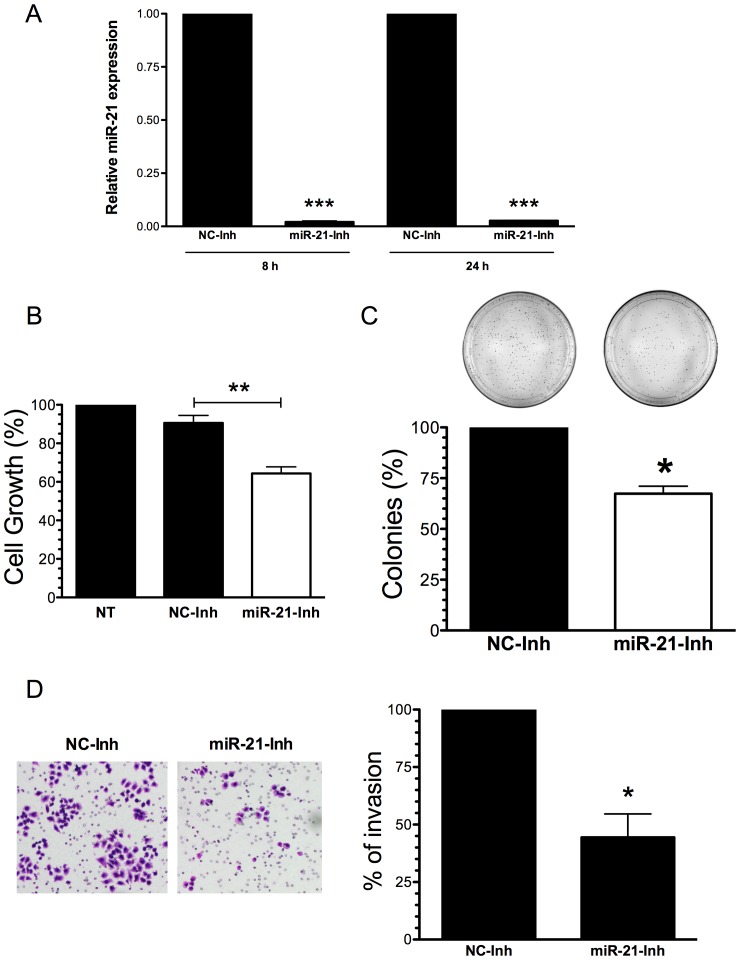
Effect of miR-21 inhibition in cell growth, proliferation and invasion. (**A**) A2780CP20 cells were transiently transfected with a miR-21 antagomir or with a negative antagomir (-) as described in the “Materials and Methods” section. Eight and 24 hours after transfection cells were collected and RNA (including miRNAs) was isolated as described in the “Materials and Methods” section. (**B**) MiR-21 inhibition reduced cell growth compared to untreated cells (NT) or with the negative control inhibitor (NC-Inh). (**C**) A2780CP20 cells were transfected as in 5A. A thousand cells were seeded in Petri dishes. Ten days later, the colonies were stained and counted. Inhibition of miR-21 decreased the ability of cells to undergo unlimited division compared with the NC-Inh. (**D**) Cell invasion was carried out as described in the “Materials and Methods” section. The number of invaded cells was expressed in percentages, taken the NC-Inh as 100%. *p<0.05, **p<0.01 compared to NC-Inh. Columns represent the means of triplicates ± S.E.M.

We next assessed the effect of miR-21 inhibition in cell growth and proliferation of A2780CP20 cells. For this set of experiments, we first confirmed the downregulation of miR-21 expression (**p<0.001) by the miR-21-Inh (Figure 5A). Comparing with a negative control miRNA inhibitor (NC-Inh), transient transfection of A2780CP20 with the miR-21 antagomir (miR-21-Inh) significantly reduced (35%, **p<0.01) cell survival (Figure 5B). MiR-21 inhibition induced long-term effects in cell growth and proliferation. In a colony formation assay, transient transfection of miR-21 inhibitor in A2780CP20 cells reduced significantly (33%, *p<0.05) the number of colonies formed compared with the NC-Inh transfected cells (Figure 5C).

Increased levels of miR-21 have been associated with accelerated metastatic rate of various tumors. Thus, we assessed whether miR-21 inhibition reduced the metastatic potential of A2780CP20 cells [16],[35],[36],[37]. Invasion assays confirmed that 50 nM of miR-21-Inh reduced significantly (50%, *p<0.05) the invasion ability of A2780CP20 cells compared with 50 nM of NC-Inh (Figure 5D). This data suggest that increased miR-21 levels are associated with the metastatic potential of cisplatin resistant ovarian cancer cells, and that miR-21 is a potential target to overcome the cisplatin resistance of highly metastatic ovarian cancer cells.

## Discussion

The major findings of this study were that the JNK-1/c-Jun pathway regulates the miR-21 expression in the cisplatin resistance of ovarian cancer cells, and that targeting miR-21 inhibited cell growth and proliferation of these cells. MiR-21, one of the most commonly upregulated miRNAs in tumor cells, is endowed with oncogenic properties, and promotes cell proliferation, invasion, and migration of cancer cell populations [11],[37],[38],[39]. MiR-21 overexpression has also been associated with drug resistance [40]. The regulation of miR-21 in cisplatin resistant ovarian cancer cells had not been well studied.

We found that the miR-21 levels correlated well with the sensitivity of ovarian cancer cells to cisplatin treatment. In addition, the total and phospho JNK-1 and c-Jun protein levels were higher in A2780CP20 compared with their parental sensitive A2780 cells. Inhibition of c-Jun phosphorylation with the JNK-1 specific inhibitor SP600125 in A2780CP20 cells decreased the pre-mir-21 mRNA and increased the PDCD4 protein levels. These effects were not observed in the cisplatin sensitive A2780 cells. Furthermore, the ChIP experiments confirmed that c-Jun binds physically to the pri-mir-21 DNA promoter regions and activates pri-mir-21 expression. In colon cancer, human promyelocytic, and in certain chemoresistant side populations of cancer stem cells, regulation of miR-21 (under different treatments) was AP-1-mediated, which is in agreement with our findings that the JNK-1/c-Jun/miR-21 pathway is activated in cisplatin resistant ovarian cancer cells.

Several miRNAs have been reported as differentially abundant in cisplatin sensitive and cisplatin resistant ovarian cancer cells [41],[42]. For example, miR-214 overexpression was associated with cisplatin resistance of ovarian cancer cells [43],[44]. Intriguing, our preliminary microarray studies (Table S1) showed lower miR-214 levels in A2780CP20 compared with A2780 cells. In other cell lines downregulation of miR-214 was associated with tumor aggressiveness [45],[46]. Thus, the miR-214 expression levels in the context of drug resistance need further analysis. Overexpression of miR-141 and downregulation of its target gene the Kelch-Like ECH-Associated Protein 1 (KEAP1) have also been associated with cisplatin resistance [47]. However, in human esophageal squamous cell carcinoma cells miR-141 appears to induce cisplatin resistance through the Yes-Associated Protein 1 (YAP1) [47],[48]. By using next generation sequencing approaches, several other differentially expressed miRNA have been identified in cisplatin sensitive and cisplatin and resistant ovarian cancer cells [41],[43],[49],[50]. However, the upstream molecular mechanism leading to dysregulation of such number of miRNAs in cisplatin resistant ovarian cancer cells remains unclear. Our study is the first showing that overexpression of miR-21 in cisplatin resistant ovarian cancer cells is a secondary event associated with the activation of the JNK-1/c-Jun pathway in these cells. This hypothesis is further supported by studies showing that the phosphorylation level of JNK-1 correlates well with progression free survival and overall survival of ovarian cancer patients [51]. Nevertheless, targeting miR-21 inhibited cell growth, proliferation and the invasion ability of cisplatin resistant cells, which demonstrates that miR-21, could be considered as a potential target to overcome the cisplatin resistance of highly metastatic ovarian cancer tumors. This idea is supported by recent reports of Chan et al whom using “The cancer Genome Atlas” (TCGA) database found that women with tumors overexpressing miR-21 exhibited shorter progression-free survival [18].

Taken together, we showed that JNK-1/c-Jun pathway is activated in cisplatin resistant ovarian cancer cells and promote miR-21 upregulation in these cells. Overexpression of miR-21, in turn activates its cognate targets, including the tumor suppressor gene, PDCD4. All of these molecular events play a pivotal role in the cisplatin resistance of ovarian cancer cells. The upstream molecular events of many other miRNAs differentially abundant in drug resistant and drug sensitive ovarian cancer cells needs to be further investigated. This could provide an explanation of how several miRNAs are dysregulated not only in ovarian cancer but also in many other tumor types.

## Supporting Information

Figure S1 **STAT3 protein levels in A2780CP20 and A2780 cells.** Protein extraction and Western blot analysis was performed as described in the “Methods” section. The STAT3 protein levels were similar in A2780CP20 and A2780 cells.
(TIFF)

Figure S2 **Effect of JNK-1 inhibition in pre-mir-21 and PDCD4 expression.** A2780 cells were treated with 10 µM SP600125 as described in the “Materials and Methods” section. RNA, protein extraction, real-time PCR and western blots were performed as described in the “Material and Methods” section. **(A)** Western blot analysis showing that treatment of A2780 cells with SP600125 did not affect the total or the phosphorylation levels of JNK-1, JNK-2/3 or c-Jun. **(B)** SYBR-I-based real-time PCR was performed to assess the relative pre-mir-21 expression levels in A2780 cells following SP600125 treatment. A small, no significant increasing, rather than a decreasing in pre-mir-21 levels was observed in SP600125-treated cells compared with DMSO-treated cells. **(C)** Western blot and densitometric analysis showed not visible changes in the PDCD4 protein levels after treatment of A2780 cells with SP600125.
(TIFF)

Figure S3 **MiR-21 promoter region.** Primers were designed to amplify a DNA region encompassing the AP-1 binding sites (grey shadows) in the miR-21 promoter region. The other pair of primers amplifies a DNA outside of the AP-1 promoter region.
(TIFF)

Figure S4 **PDCD4 expression in miR-21 and empty vector clones.** Protein extraction and Western blot analysis was performed as described in the “Methods” section. A2780-EV: empty vector clones. A2780-miR-21: miR-21 overexpressed clones.
(TIFF)

Figure S5 **Visualization of the pre-miR-21 stable transfection in A2780 cells.** Stable transected A2780 cells were was monitored by the tGFP signal. Red: GFP. Blue: DAPI.
(TIFF)

Table S1 **Microarray data analysis.** Two way-ANOVA was used to calculate genes with fold changes of +/− 3 in A2780CP20 vs. A2780 cells.
(PDF)

Table S2 **List of primers used in this study.**
(PDF)
