# Correction: Upregulation of miR-21 in Cisplatin Resistant Ovarian Cancer via JNK-1/c-Jun Pathway

**DOI:** 10.1371/journal.pone.0116447

**Published:** 2014-12-26

**Authors:** The PLOS ONE Staff

The following information is missing from the Funding section: Infrastructure support: RCMI: G12RR03051, G12MD007600. The correct funding information is as follows: Funding: This project was supported partially by the NIH/NCI (1K22CA166226-01A1) to PEVM, institutional seed funds from the University of Puerto Rico. Comprehensive Cancer Center (PEVM), National Institute of Health, and Minority Biomedical Research Support (MBRS) RISE Grant Number R25-GM061838 (IEV). Infrastructure support: RCMI: G12RR03051, G12MD007600. The funders had no role in study design, data collection and analysis, decision to publish, or preparation of the manuscript.

## References

[1] Echevarría-Vargas IM, Valiyeva F, Vivas-Mejía PE (2014) Upregulation of miR-21 in Cisplatin Resistant Ovarian Cancer via JNK-1/c-Jun Pathway. PLoS ONE 9(5): e97094 doi:10.1371/journal.pone.0097094 24865582 10.1371/journal.pone.0097094 PMC4035252

