# Supplementary figures and images for: Upregulation of miR-21 in Cisplatin Resistant Ovarian Cancer via JNK-1/c-Jun Pathway
Source: PLoS One. 2014 May 27;9(5):e97094. doi: 10.1371/journal.pone.0097094 (PMC4035252; doi:10.1371/journal.pone.0097094)

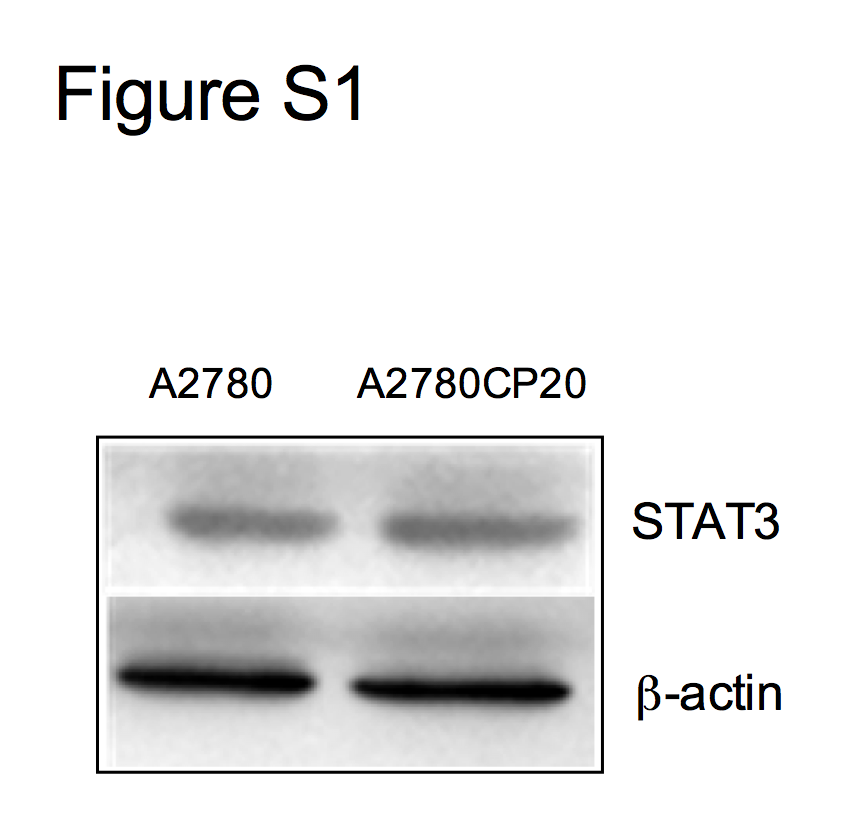

Supplement: Figure S1 — STAT3 protein levels in A2780CP20 and A2780 cells. Protein extraction and Western blot analysis was performed as described in the “Methods” section. The STAT3 protein levels were similar in A2780CP20 and A2780 cells. (TIFF) [file pone.0097094.s001.tiff]

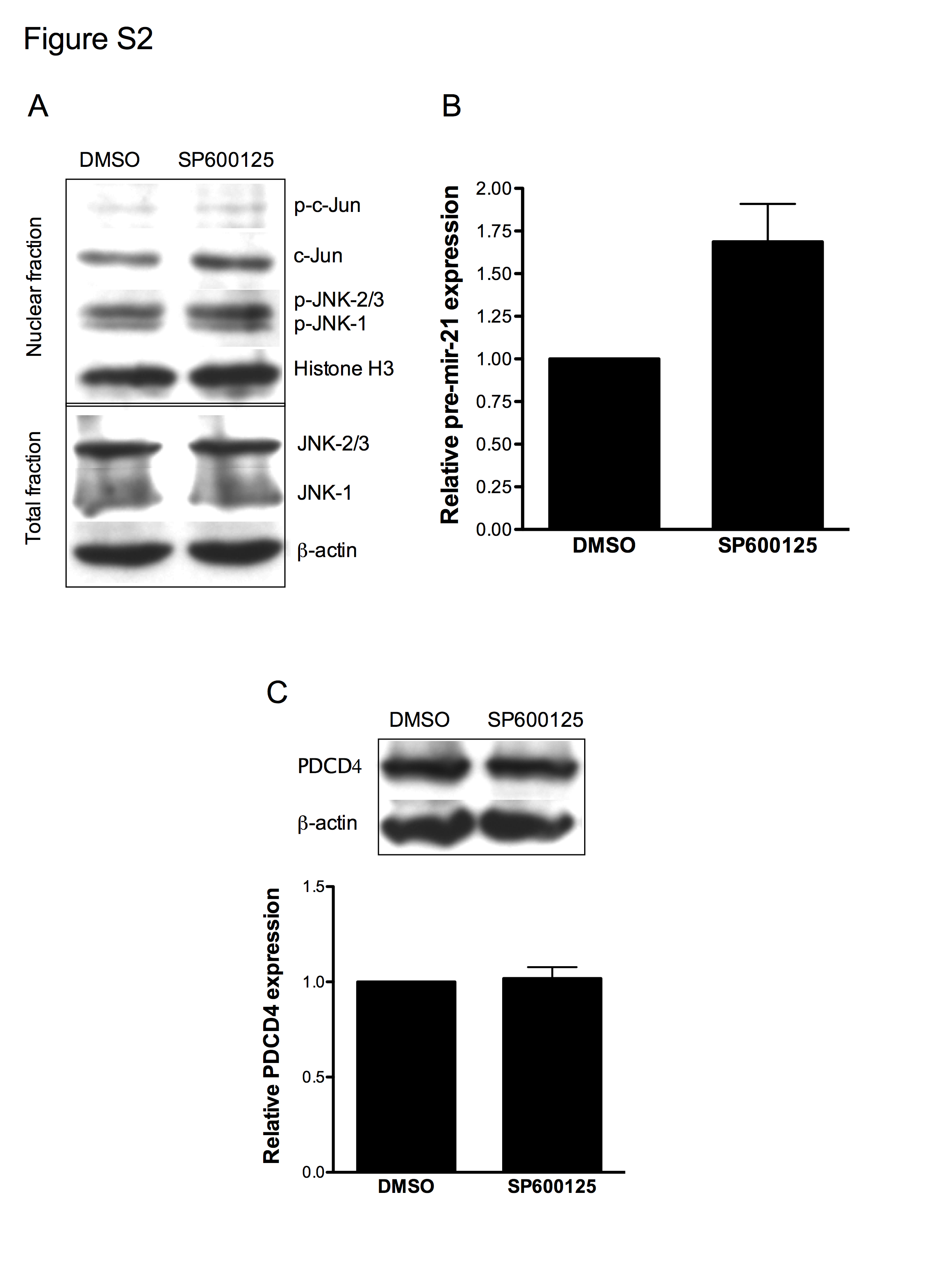

Supplement: Figure S2 — Effect of JNK-1 inhibition in pre-mir-21 and PDCD4 expression. A2780 cells were treated with 10 µM SP600125 as described in the “Materials and Methods” section. RNA, protein extraction, real-time PCR and western blots were performed as described in the “Material and Methods” section. (A) Western blot analysis showing that treatment of A2780 cells with SP600125 did not affect the total or the phosphorylation levels of JNK-1, JNK-2/3 or c-Jun. (B) SYBR-I-based real-time PCR was performed to assess the relative pre-mir-21 expression levels in A2780 cells following SP600125 treatment. A small, no significant increasing, rather than a decreasing in pre-mir-21 levels was observed in SP600125-treated cells compared with DMSO-treated cells. (C) Western blot and densitometric analysis showed not visible changes in the PDCD4 protein levels after treatment of A2780 cells with SP600125. (TIFF) [file pone.0097094.s002.tiff]

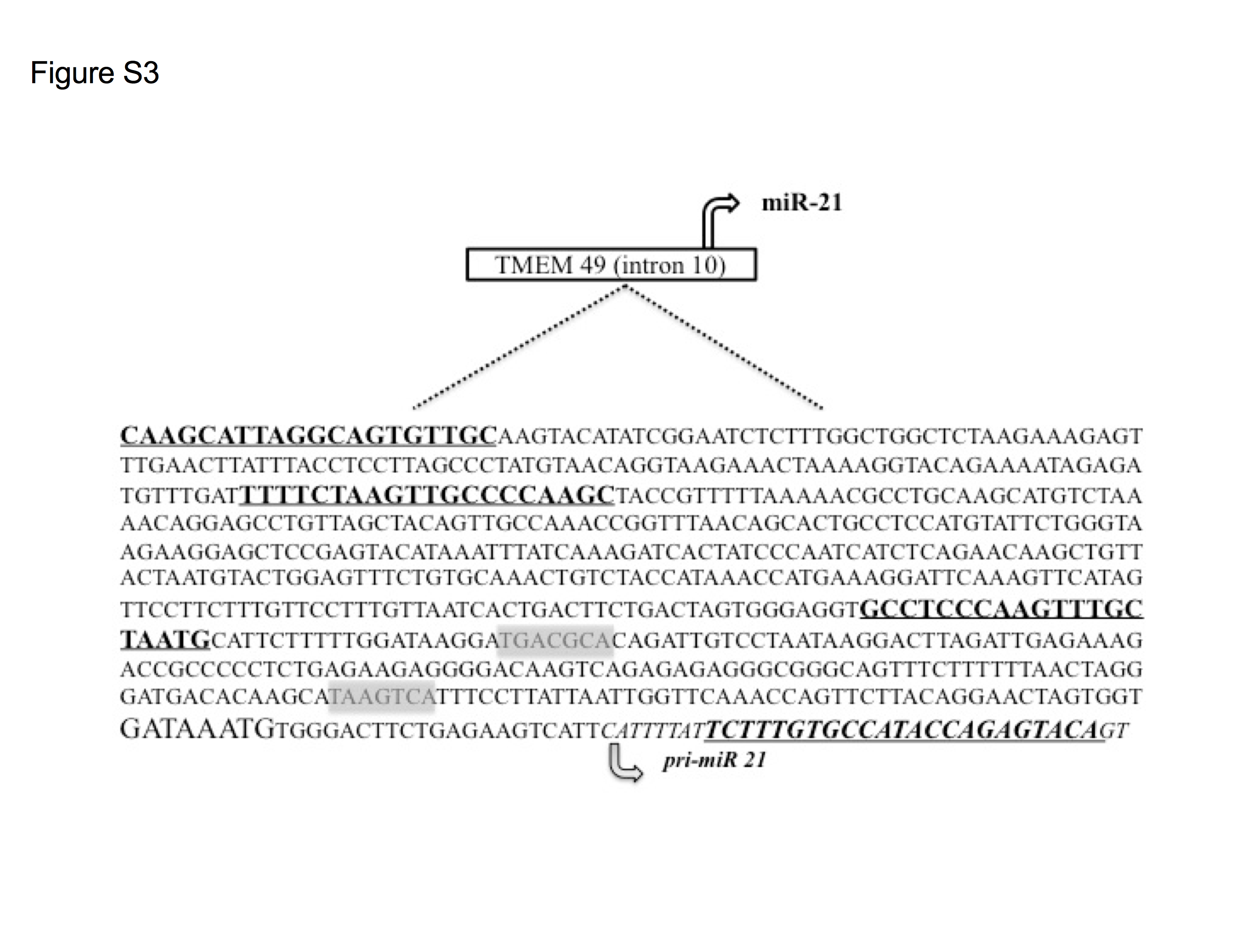

Supplement: Figure S3 — MiR-21 promoter region. Primers were designed to amplify a DNA region encompassing the AP-1 binding sites (grey shadows) in the miR-21 promoter region. The other pair of primers amplifies a DNA outside of the AP-1 promoter region. (TIFF) [file pone.0097094.s003.tiff]

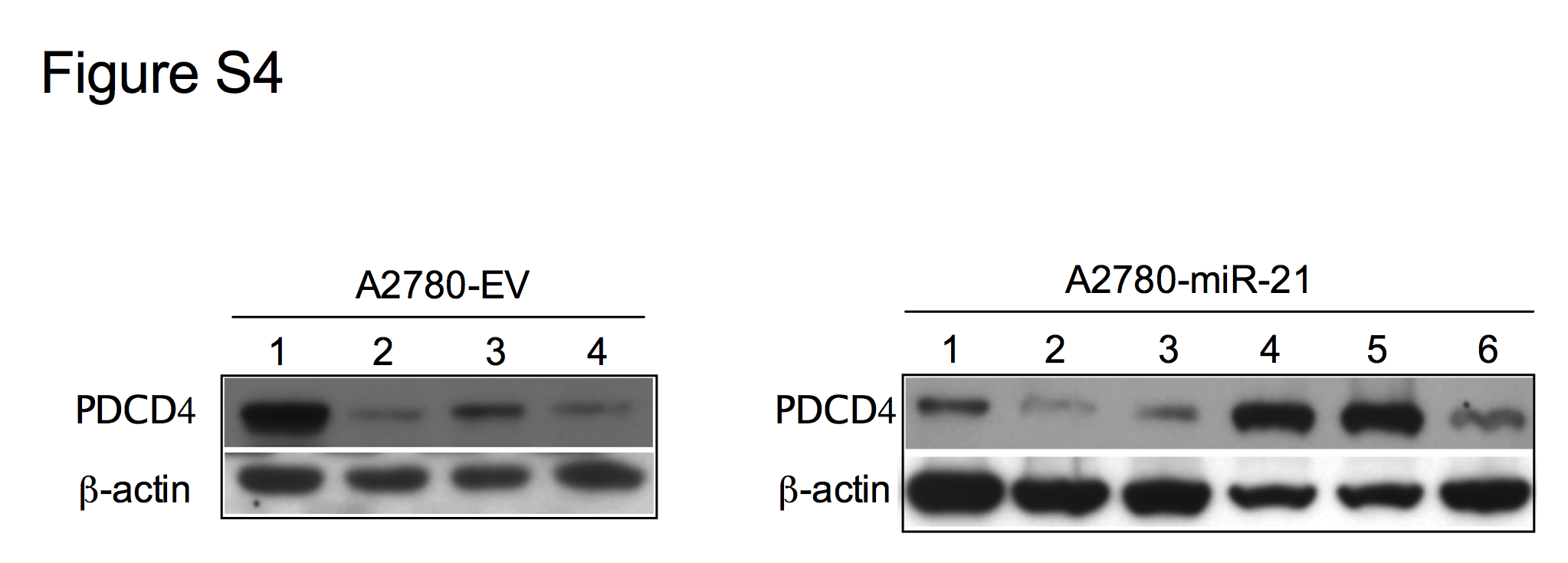

Supplement: Figure S4 — PDCD4 expression in miR-21 and empty vector clones. Protein extraction and Western blot analysis was performed as described in the “Methods” section. A2780-EV: empty vector clones. A2780-miR-21: miR-21 overexpressed clones. (TIFF) [file pone.0097094.s004.tiff]

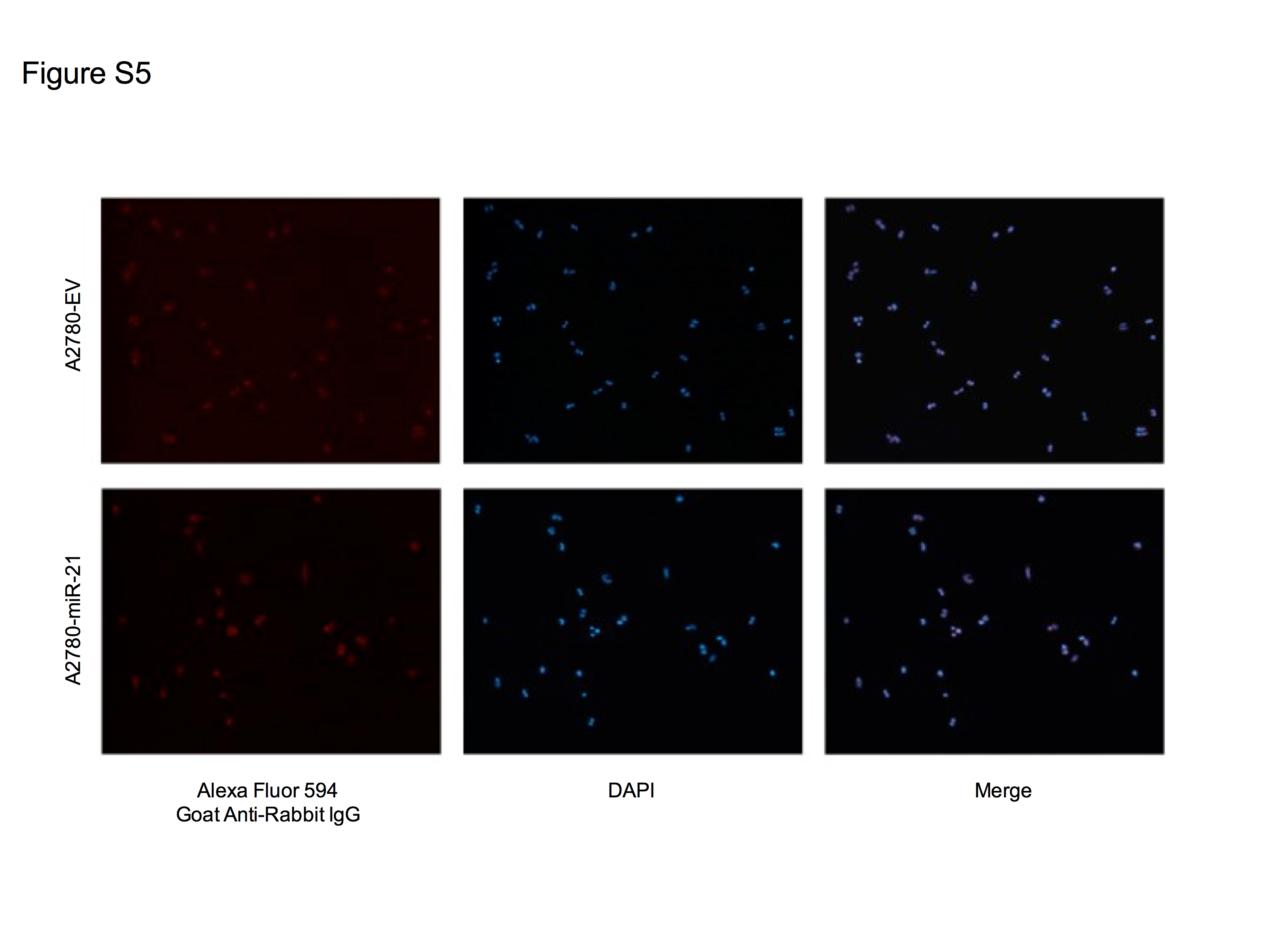

Supplement: Figure S5 — Visualization of the pre-miR-21 stable transfection in A2780 cells. Stable transected A2780 cells were was monitored by the tGFP signal. Red: GFP. Blue: DAPI. (TIFF) [file pone.0097094.s005.tiff]
