# Supplementary material for: Upregulation of miR-21 in Cisplatin Resistant Ovarian Cancer via JNK-1/c-Jun Pathway
Source: PLoS One. 2014 May 27;9(5):e97094. doi: 10.1371/journal.pone.0097094 (PMC4035252; doi:10.1371/journal.pone.0097094)
Supplement: Table S1 — Microarray data analysis. Two way-ANOVA was used to calculate genes with fold changes of +/− 3 in A2780CP20 vs. A2780 cells. (PDF) [file pone.0097094.s006.pdf]

Table S1

Gene Assignment	Gene Symbol	Reference Sequence	Fold-Change
**Fold Change >3**			
mucin 15, cell surface associated 11p14.3	MUC15	NM_001135091	165.421
claudin 1	CLDN1	NM_021101	127.116
ATPase, Na+/K+ transporting, beta 1 polypeptide	ATP1B1	NM_001677	116.97
ankyrin repeat domain 1 (cardiac muscle)	ANKRD1	NM_014391	76.1093
ISL LIM homeobox 1	ISL1	NM_002202	59.7141
pleckstrin and Sec7 domain containing 3	PSD3	NM_015310	56.8859
claudin 16	CLDN16	NM_006580	51.0911
leprecan-like 1	LEPREL1	NM_018192	50.7382
pleckstrin and Sec7 domain containing 3	PSD3	NM_015310	45.0983
sodium channel, voltage-gated, type IX, alpha subunit	SCN9A	NM_002977	42.5179
leucine rich repeat transmembrane neuronal 4	LRRTM4	NM_024993	41.0696
Rho GTPase activating protein 28	ARHGAP28	NM_001010000	39.3966
SH2 domain containing 4A	SH2D4A	NM_022071	33.0139
matrix metallopeptidase 3 (stromelysin 1, progelatinase)	MMP3	NM_002422	32.1111
anoctamin 3	ANO3	NM_031418	30.1691
zinc finger protein 204 (pseudogene)	ZNF204P	NR_002722	25.0198
G protein-coupled receptor 126	GPR126	NM_020455	22.7848
transmembrane 4 L six family member 1	TM4SF1	NM_014220	22.0087
potassium inwardly-rectifying channel, subfamily J, member	KCNJ2	NM_000891	21.5557
oligonucleotide/oligosaccharide-binding fold containin	OBFC2A	NM_001031716	19.7667
transient receptor potential cation channel, subfamily C,	TRPC4	NM_016179	19.1597
CD36 molecule (thrombospondin receptor)	CD36	NM_001001548	18.8959
neuregulin 1	NRG1	NM_013958	18.507
annexin A1	ANXA1	NM_000700	18.3792
tumor necrosis factor receptor superfamily, member 19	TNFRSF19	NM_148957	18.1261
protein kinase, AMP-activated, alpha 2 catalytic subunit	PRKAA2	NM_006252	17.1484

interleukin 1 receptor accessory protein	IL1RAP	NM_002182	16.971
N-myc (and STAT) interactor	NMI	NM_004688	15.8345
potassium inwardly-rectifying channel, subfamily J, member	KCNJ16	NM_170742	14.6213
lysophosphatidic acid receptor 1	LPAR1	NM_057159	14.2709
galactosylceramidase	GALC	NM_000153	13.737
fibronectin leucine rich transmembrane protein 3	FLRT3	NM_198391	13.737
Sp3 transcription factor pseudogene	SP3P	BC036697	13.2691
sodium channel, voltage-gated, type I, alpha subunit	SCN1A	NM_001165963	13.0412
guanylate binding protein 1, interferon-inducible, 67kDa	GBP1	NM_002053	12.9511
protein kinase C	PRKCH	NM_006255	12.7728
enolase 2 (gamma, neuronal)	ENO2	NM_001975	12.5533
latrophilin 2	LPHN2	NM_012302	12.4666
zinc finger protein 82 homolog (mouse)	ZFP82	NM_133466	12.0003
epithelial cell adhesion molecule	EPCAM	NM_002354	11.8351
myosin IB	MYO1B	NM_001130158	11.7942
solute carrier family 6 (neutral amino acid transporter)	SLC6A15	NM_182767	11.4716
RNA binding motif protein 24	RBM24	NM_001143942	11.353
cysteine-rich secretory protein LCCL domain containing	CRISPLD1	NM_031461	11.1193
paired box 6	PAX6	NM_000280	11.0809
periostin, osteoblast specific factor	POSTN	NM_006475	11.0809
ankyrin repeat domain 5	ANKRD5	NM_022096	10.9663
microRNA 622	MIR622	NR_030754	10.7034
phosphoinositide-3-kinase, catalytic, alpha polypeptide	PIK3CA	NM_006218	10.5561
synaptotagmin XIV	SYT14	NR_027458	10.3747
ATP-binding cassette, sub-family B (MDR/TAP), member 1	ABCB1	NM_000927	10.3747
RAB3A interacting protein (rabin3)	RAB3IP	NM_175623	9.57983
beta-1,3-N-acetylgalactosaminyltransferase 1	B3GALNT1	NM_001038628	9.44794
POU class 1 homeobox 1	POU1F1	NM_001122757	8.81524
aldehyde dehydrogenase 1 family, member A1	ALDH1A1	NM_000689	8.69388
solute carrier family 39 (zinc transporter), member 8	SLC39A8	NM_022154	8.54452

fibrillin 1	FBN1	NM_000138	8.4855
myristoylated alanine-rich protein kinase C substrate	MARCKS	NM_002356	8.36868
phospholipase A2, group XVI	PLA2G16	NM_007069	8.33973
asparaginase like 1	ASRGL1	NM_001083926	8.22491
serpin peptidase inhibitor, clade B (ovalbumin), member	SERPINB1	NM_030666	8.1681
matrix metallopeptidase 1 (interstitial collagenase)	MMP1	NM_002421	8.05564
androgen-induced 1	AIG1	NM_016108	8
matrix metallopeptidase 16 (membrane-inserted)	MMP16	AL136588	7.94474
transmembrane protein 30B	TMEM30B	NM_001017970	7.88986
target of myb1-like 2 (chicken)	TOM1L2	NM_001082968	7.80825
myoferlin	MYOF	NM_013451	7.59474
UDP-N-acetyl-alpha-D-galactosamine:polypeptide N-acetylg	GALNT13	NM_052917	7.51618
DnaJ (Hsp40) homolog, subfamily C, member 15	DNAJC15	NM_013238	7.46426
guanylate cyclase 1, soluble, alpha 3	GUCY1A3	NM_000856	7.4127
transmembrane protein 56	TMEM56	NM_152487	7.3615
CD55 molecule, decay accelerating factor for complement	CD55	NM_001114752	7.31065
microsomal glutathione S-transferase 1	MGST1	NM_145792	7.26015
small VCP/p97-interacting protein	SVIP	NM_148893	7.21
sel-1 suppressor of lin-12-like 3 (C. elegans) 2	SEL1L3	NM_015187	7.1602
laminin, alpha 2	LAMA2	NM_000426	7.03719
matrix metallopeptidase 16 (membrane-inserted)	MMP16	NM_005941	6.89237
filamin A interacting protein 1	FILIP1	NM_015687	6.86852
tetratricopeptide repeat domain 39A	TTC39A	NM_001144832	6.77396
poly (ADP-ribose) polymerase family, member 8	PARP8	NM_001178055	6.77396
serine/threonine kinase 33	STK33	NM_030906	6.75053
opioid growth factor receptor-like 1	OGFRL1	NM_024576	6.65759
solute carrier family 16, member 6	SLC16A6	NM_001174166	6.52058
solute carrier family 41, member 2	SLC41A2	NM_032148	6.40856
sulfatase 1	SULF1	NM_001128205	6.29846
synovial sarcoma, X breakpoint 2 interacting protein	SSX2IP	NM_014021	6.29846

cingulin-like 1	CGNL1	NM_032866	6.21175
serpin peptidase inhibitor, clade B (ovalbumin), member	SERPINB9	NM_004155	6.1475
TIMP metallopeptidase inhibitor 1	TIMP1	NM_003254	5.91755
solute carrier family 27 (fatty acid transporter), member	SLC27A2	NM_003645	5.89708
tandem C2 domains, nuclear	TC2N	NM_001128596	5.77572
CAP-GLY domain containing linker protein family, member 4	CLIP4	NM_024692	5.75573
tumor necrosis factor receptor superfamily, member 10d	TNFRSF10D	NM_003840	5.52127
heparan sulfate 6-O-sulfotransferase 2	HS6ST2	NM_001077188	5.46416
leucine rich repeat neuronal 3	LRRN3	NM_001099660	5.46416
epithelial cell adhesion molecule	EPCAM	NM_002354	5.40764
StAR-related lipid transfer (START) domain containing 4	STARD4	NM_139164	5.37029
MORC family CW-type zinc finger 4	MORC4	NM_024657	5.29636
molybdenum cofactor sulfurase	MOCOS	NM_017947	5.24157
adaptor-related protein complex 1, sigma 3 subunit	AP1S3	NM_001039569	5.22344
family with sequence similarity 26, member D	FAM26D	NM_153036	5.22344
cellular retinoic acid binding protein 2	CRABP2	NM_001878	5.18736
matrix metallopeptidase 10 (stromelysin 2)	MMP10	NM_002425	5.15153
arginase, type II	ARG2	NM_001172	5.0806
microRNA 27b	MIR27B	NR_029665	5.0806
exoribonuclease 1	ERI1	NM_153332	5.0806
AHNAK nucleoprotein	AHNAK	NM_001620	5.0806
collagen, type XI, alpha 1	COL11A1	NM_001854	4.99332
solute carrier family 16, member 9	SLC16A9	NM_194298	4.99332
arrestin domain containing 3	ARRDC3	NM_020801	4.97605
regulator of G-protein signaling 20	RGS20	NM_170587	4.95883
nucleosome assembly protein 1-like 2	NAP1L2	NM_021963	4.80654
solute carrier family 9 (sodium/hydrogen exchanger)	SLC9A7	NM_032591	4.70763
chemokine (C-X-C motif) ligand 1	CXCL1	NM_001511	4.67511
cysteinyl leukotriene receptor 2	CYSLTR2	NM_020377	4.67511
fibroblast growth factor 2 (basic)	FGF2	NM_002006	4.64282

family with sequence similarity 46, member A	FAM46A	NM_017633	4.5789
transgelin	TAGLN	NM_001001522	4.5789
SAM domain and HD domain 1	SAMHD1	NM_015474	4.54727
zinc finger protein 431	ZNF431	NM_133473	4.53154
solute carrier family 16, member 14	SLC16A14	NM_152527	4.48466
SIX homeobox 1	SIX1	NM_005982	4.46915
phosphorylase, glycogen, liver	PYGL	NM_002863	4.45369
chromosome 6 open reading frame 192	C6orf192	NM_052831	4.45369
ectonucleotide pyrophosphatase/phosphodiesterase 1	ENPP1	NM_006208	4.43828
sulfide quinone reductase-like (yeast)	SQRDL	NM_021199	4.40762
matrix metallopeptidase 12 (macrophage elastase)	MMP12	NM_002426	4.37717
zinc finger protein 620	ZNF620	NM_175888	4.37717
phosphate cytidylyltransferase 1, choline, beta	PCYT1B	NM_004845	4.34694
MOCO sulphurase C-terminal domain containing 1	MOSC1	NM_022746	4.3319
jun oncogene	JUN	NM_002228	4.31691
sterile alpha motif domain containing 12	SAMD12	NM_207506	4.27226
guanine nucleotide binding protein (G protein)i	GNAI1	NM_002069	4.27226
UDP-Gal:betaGlcNAc beta 1,3-galactosyltransferase	B3GALT1	NM_020981	4.21344
EPH receptor A5	EPHA5	NM_004439	4.21344
cofilin 2 (muscle)	CFL2	NM_021914	4.19887
ATP-binding cassette, sub-family A (ABC1), member 5	ABCA5	NM_018672	4.19887
phospholipase A2, group IVA (cytosolic, calcium-dependent)	PLA2G4A	NM_024420	4.16986
myosin VI	MYO6	NM_004999	4.14106
5'-nucleotidase, cytosolic II	NT5C2	NM_012229	4.12673
leukemia inhibitory factor (cholinergic differentiation factor)	LIF	NM_002309	4.09823
sprouty homolog 2 (Drosophila)	SPRY2	NM_005842	4.06992
transmembrane protein 62	TMEM62	NM_024956	4.06992
UDP-N-acetyl-alpha-D-galactosamine	GALNT14	NM_024572	4.06992
Cbp/p300-interacting transactivator	CITED2	NM_006079	4.06992
ankylosis, progressive homolog (mouse)	ANKH	NM_054027	4.02782

retinol dehydrogenase 10 (all-trans)	RDH10	NM_172037	4.02782
activating signal cointegrator 1 complex subunit 1	ASCC1	NM_015947	4
myelin expression factor 2	MYEF2	NM_016132	3.98616
pigeon homolog (Drosophila)	PION	NM_017439	3.97237
glycine amidinotransferase	GATM	NM_001482	3.94493
apolipoprotein B mRNA editing enzyme	APOBEC3B	NM_004900	3.93128
chemokine (C-X-C motif) ligand 16	CXCL16	NM_022059	3.90413
elongation of very long chain fatty acids	ELOVL4	NM_022726	3.89062
protein tyrosine phosphatase	PTPN3	NM_002829	3.82378
hepatocyte nuclear factor 4, gamma	HNF4G	NM_004133	3.78423
F11 receptor	F11R	NM_016946	3.78423
src kinase associated phosphoprotein 1	SKAP1	NM_003726	3.78423
zinc finger, matrin type 3	ZMAT3	NM_022470	3.77114
PRELI domain containing 2	PRELID2	NM_182960	3.75809
interleukin 17 receptor B	IL17RB	NM_018725	3.75809
elongation factor, RNA polymerase II	ELL2	NM_012081	3.70635
plastin 1	PLS1	NM_001172312	3.70635
ribosomal protein L22-like 1	RPL22L1	NM_001099645	3.68075
proteasome (prosome, macropain) subunit, beta type, 9	PSMB9	NM_002800	3.66802
SP140 nuclear body protein-like	SP140L	NM_138402	3.65533
microRNA 21	MIR21	NR_029493	3.65533
SH3-domain GRB2-like endophilin B1	SH3GLB1	NM_016009	3.64268
schwannomin interacting protein 1	SCHIP1	NM_014575	3.61752
tetratricopeptide repeat domain 39B	TTC39B	NM_152574	3.5801
dedicator of cytokinesis 5	DOCK5	NM_024940	3.54307
methionine adenosyltransferase II	MAT2B	NM_013283	3.50642
galactosidase, alpha	GLA	NM_000169	3.47015
protein tyrosine phosphatase	PTPRK	NM_001135648	3.45815
X-linked Kx blood group (McLeod syndrome)	XK	NM_021083	3.44618
heparanase	HPSE	NM_006665	3.42238

phorbol-12-myristate-13-acetate-induced protein 1	PMAIP1	NM_021127	3.41054
mitogen-activated protein kinase kinase kinase 2	MAP3K2	NM_006609	3.39874
zinc finger protein 350	ZNF350	NM_021632	3.38698
lectin, galactoside-binding, soluble, 8	LGALS8	NM_006499	3.38698
protein tyrosine phosphatase, receptor type, G	PTPRG	NM_002841	3.36359
melanocortin 2 receptor accessory protein 2	MRAP2	NM_138409	3.35195
family with sequence similarity 111, member A	FAM111A	NM_022074	3.35195
arrestin domain containing 4	ARRDC4	NM_183376	3.31728
ATP synthase mitochondrial F1 complex assembly factor 2	ATPAF2	NM_145691	3.28297
interferon-related developmental regulator 1	IFRD1	NM_001550	3.28297
sorting nexin 10	SNX10	NM_013322	3.27161
SH3-domain kinase binding protein 1	SH3KBP1	NM_031892	3.22657
l(3)mbt-like 4 (Drosophila)	L3MBTL4	NM_173464	3.22657
contactin associated protein-like 3	CNTNAP3	NM_033655	3.2154
ubiquitin specific peptidase 18	USP18	NM_017414	3.2154
katanin p60 subunit A-like 1	KATNAL1	NM_001014380	3.20428
tuftelin 1	TUFT1	NM_020127	3.19319
chromatin modifying protein 2B	CHMP2B	NM_014043	3.18215
sushi, von Willebrand factor type A, EGF and pentraxin domain	SVEP1	NM_153366	3.14923
RAD9 homolog B (S. pombe)	RAD9B	NM_152442	3.11666
transporter 1, ATP-binding cassette, sub-family B (MDR/TAP)	TAP1	NM_000593	3.08442
coiled-coil domain containing 109B	CCDC109B	NM_017918	3.07375
caspase 6, apoptosis-related cysteine peptidase	CASP6	NM_001226	3.06312
interleukin-1 receptor-associated kinase 4	IRAK4	NM_001114182	3.05252
syntaxin 7	STX7	NM_003569	3.04196
leucine rich repeat containing 1	LRRC1	NM_018214	3.02095
Ca++-dependent secretion activator 2	CADPS2	NM_017954	3.00008
**>3-fold downregulation**			
poly (ADP-ribose) polymerase family, member 14	PARP14	NM_017554	3.00008
TBC1 domain family, member 4	TBC1D4	NM_014832	-3.00008

quinolinate phosphoribosyltransferase	QPRT	NM_014298	-3.03143
zinc finger protein 516	ZNF516	NM_014643	-3.03143
fibroblast growth factor receptor 4	FGFR4	NM_213647	-3.03143
transmembrane protein 47	TMEM47	NM_031442	-3.08442
sterile alpha motif domain containing 4A	SAMD4A	NM_015589	-3.09513
glycosyltransferase 8 domain containing 2	GLT8D2	NM_031302	-3.09513
inositol 1,4,5-triphosphate receptor, type 2	ITPR2	NM_002223	-3.12748
ring finger protein 207	RNF207	NM_207396	-3.13834
junctophilin 3	JPH3	NM_020655	-3.2154
twist homolog 1 (Drosophila)	TWIST1	NM_000474	-3.32879
KIAA0355	KIAA0355	NM_014686	-3.34035
UDP-glucose ceramide glucosyltransferase	UGCG	NM_003358	-3.35195
mannose receptor, C type 2	MRC2	NM_006039	-3.38698
leukocyte receptor tyrosine kinase	LTK	NM_002344	-3.42238
ankyrin 3, node of Ranvier (ankyrin G)	ANK3	NM_020987	-3.43426
ankyrin repeat and LEM domain containing 1	ANKLE1	NM_152363	-3.47015
kin of IRRE like (Drosophila)	KIRREL	NM_018240	-3.47015
programmed cell death 4	PDCD4	NM_145341	-3.5186
tetratricopeptide repeat, ankyrin repeat and coiled-coil c	TANC2	NM_025185	-3.53081
glutamate receptor, metabotropic 1	GRM1	NM_001114329	-3.54307
protein tyrosine phosphatase, receptor type, S	PTPRS	NM_002850	-3.56771
leucine rich repeat containing 4C	LRRC4C	NM_020929	-3.5801
NADPH oxidase 3	NOX3	NM_015718	-3.605
suppression of tumorigenicity 5	ST5	NM_005418	-3.61752
microRNA 214	MIR214	NR_029627	-3.65533
nuclear factor of activated T-cells, cytoplasmic, calcine	NFATC1	NM_172387	-3.66802
fibroblast activation protein, alpha	FAP	NM_004460	-3.78423
checkpoint with forkhead and ring finger domains	CHFR	NM_001161344	-3.81055
diacylglycerol kinase, beta 90kDa	DGKB	NM_004080	-3.82378
leukocyte-associated immunoglobulin-like receptor 1	LAIR1	NM_002287	-3.85038

breast carcinoma amplified sequence 3	BCAS3	NM_001099432	-3.86375
glypican 6	GPC6	NM_005708	-4.01389
C1q and tumor necrosis factor related protein 3	C1QTNF3	NM_181435	-4.04181
FRAS1 related extracellular matrix protein 2	FREM2	NM_207361	-4.06992
ribosomal modification protein rimK-like family member B	RIMKLB	NM_020734	-4.06992
creatine kinase, brain	CKB	NM_001823	-4.08405
regulator of G-protein signaling 16	RGS16	NM_002928	-4.14106
matrix-remodelling associated 8	MXRA8	NM_032348	-4.16986
dynein, axonemal, heavy chain 7	DNAH7	NM_018897	-4.22807
fibulin 1	FBLN1	NM_006486	-4.42292
follistatin-like 1	FSTL1	NM_007085	-4.50023
PERP, TP53 apoptosis effector	PERP	NM_022121	-4.5789
fibroblast growth factor 18	FGF18	NM_003862	-4.61075
WNT inhibitory factor 1	WIF1	NM_007191	-4.69134
transcription elongation factor A (SII)-like 8	TCEAL8	NM_153333	-4.78991
ATP-binding cassette, sub-family D (ALD), member 2	ABCD2	NM_005164	-4.92458
Meis homeobox 2	MEIS2	NM_172316	-4.97605
MID1 interacting protein 1	MID1IP1	NM_021242	-5.06303
phosphatidylinositol transfer protein, cytoplasmic 1	PITPNC1	NM_181671	-5.06303
glutamate receptor, ionotropic, kainate 2	GRIK2	NM_175768	-5.06303
histone cluster 1	HIST1H3F	NM_021018	-5.11594
Nik related kinase	NRK	NM_198465	-5.18736
serpin peptidase inhibitor	SERPING1	NM_000062	-5.18736
family with sequence similarity 5, member C	FAM5C	NM_199051	-5.25977
v-erb-a erythroblastic leukemia viral oncogene homolog 4	ERBB4	NM_005235	-5.31474
transmembrane protein 195	TMEM195	NM_001004320	-5.33319
vascular cell adhesion molecule 1	VCAM1	NM_001078	-5.33319
calcium channel, voltage-dependent, gamma subunit 7	CACNG7	NM_031896	-5.38893
EMI domain containing 2	EMID2	NM_133457	-5.44526
glypican 5	GPC5	NM_004466	-5.59834

lin-28 homolog A (C. elegans)	LIN28A	NM_024674	-5.65685
solute carrier family 8	SLC8A1	NM_021097	-5.67649
homeobox C12	HOXC12	NM_173860	-5.71598
transient receptor potential cation channel, subfamily A	TRPA1	NM_007332	-5.89708
homeobox C13	HOXC13	NM_017410	-6.12623
metallophosphoesterase domain containing 2	MPPED2	NM_001584	-6.19026
hydroxysteroid (17-beta) dehydrogenase 14	HSD17B14	NM_016246	-6.19026
histone cluster 1	HIST1H3J	NM_003535	-6.32033
neuropilin 2	NRP2	NM_201266	-6.36429
sema domain, transmembrane domain (TM), and cytoplasmic domain	SEMA6A	NM_020796	-6.47554
chromosome 8 open reading frame 4	C8orf4	NM_020130	-6.47554
carboxypeptidase A2 (pancreatic)	CPA2	NM_001869	-6.49802
SLIT and NTRK-like family, member 3	SLITRK3	NM_014926	-7.21
heparan sulfate (glucosamine) 3-O-sulfotransferase 3B1	HS3ST3B1	NM_006041	-7.43844
membrane metallo-endopeptidase	MME	NM_007288	-7.46426
syntaxin binding protein 5 (tomosyn)	STXBP5	NM_001127715	-7.6211
potassium large conductance calcium-activated channel	KCNMA1	NM_001014797	-7.64756
nestin	NES	NM_006617	-7.67411
phospholipase B domain containing 1	PLBD1	NM_024829	-7.80825
CD99 molecule	CD99	NM_002414	-7.86256
decorin	DCN	NM_001920	-8.02777
aldehyde dehydrogenase 2 family (mitochondrial)	ALDH2	NM_000690	-8.08361
ST6 beta-galactosamide alpha-2,6-sialyltranferase 2	ST6GAL2	NM_032528	-8.28212
latrophilin 3	LPHN3	NM_015236	-8.54452
chromosome 6 open reading frame 218	C6orf218	NR_027793	-8.6638
sterile alpha motif domain containing 5	SAMD5	NM_001030060	-8.78474
transmembrane protease, serine 15	TMPRSS15	NM_002772	-9.12611
nucleosome assembly protein 1-like 3	NAP1L3	NM_004538	-9.54669
integral membrane protein 2B	ITM2B	NM_021999	-10.0561

single-stranded DNA binding protein 2	SSBP2	NM_012446	-10.2319
MORC family CW-type zinc finger 1	MORC1	NM_014429	-10.9283
interferon induced with helicase C domain 1	IFIH1	NM_022168	-11.0425
cytochrome b5 type A (microsomal)	CYB5A	NM_148923	-11.1579
dehydrogenase/reductase (SDR family) member 2	DHRS2	NM_182908	-11.1967
ubiquitin carboxyl-terminal esterase L1	UCHL1	NM_004181	-11.353
zinc finger protein, multitype 2	ZFPM2	NM_012082	-11.7533
collagen, type III, alpha 1	COL3A1	NM_000090	-11.9588
EPH receptor A7	EPHA7	NM_004440	-12.3377
solute carrier family 30 (zinc transporter), member 8	SLC30A8	NM_001172813	-12.3805
homeobox C11	HOXC11	NM_014212	-12.3805
protocadherin 10	PCDH10	NM_032961	-12.8171
transcription factor AP-2 alpha	TFAP2A	NM_003220	-13.6422
forkhead box G1	FOXG1	NM_005249	-13.8806
protein tyrosine phosphatase, receptor type, D	PTPRD	NM_002839	-14.1723
lysyl oxidase	LOX	NM_002317	-14.4701
calbindin 1, 28kDa	CALB1	NM_004929	-14.5707
protocadherin 7	PCDH7	NM_032456	-14.723
EPH receptor A3	EPHA3	NM_005233	-15.6165
protein kinase, cGMP-dependent, type I	PRKG1	NM_001098512	-16.1672
tissue factor pathway inhibitor 2	TFPI2	NM_006528	-17.2677
EGF-like repeats and discoidin I-like domains 3	EDIL3	NM_005711	-20.182
procollagen C-endopeptidase enhancer	PCOLCE	NM_002593	-23.4254
protocadherin 20	PCDH20	NM_022843	-24.6754
transmembrane protein 88	TMEM88	NM_203411	-26.2637
cadherin 10, type 2 (T2-cadherin)	CDH10	NM_006727	-26.8156
