# Supplementary material for: Upregulation of miR-21 in Cisplatin Resistant Ovarian Cancer via JNK-1/c-Jun Pathway
Source: PLoS One. 2014 May 27;9(5):e97094. doi: 10.1371/journal.pone.0097094 (PMC4035252; doi:10.1371/journal.pone.0097094)
Supplement: Table S2 — List of primers used in this study. (PDF) [file pone.0097094.s007.pdf]

Table S2

Experiment	Primer	Sequence 5'-3'
Primer extension	PDCD4_F	AATAAGCGCCGCCCTCTCGC
	PDCD4_R	TGCTCGCCCAGGTTCCTGGT
	TMEM 49_F[20]	GTCGGAGCGGCTCCTCAAGAGTT
	TMEM 49_R[20]	CTTTGACGATGCCATAATTTTGA
	Jun_F	CCCCAAGATCCTGAAACAGA
	Jun_R	CCGTTGCTGGACTGGATTAT
qRT-PCR	Pre-miR-21_F[17]	CATTGTGGGTTTTGAAAAGGT
	Pre-miR-21_R[17]	CCACGACTAGAGGCTGACTTAGA
	Actin_F	CCCTTTTTGTCCCCCAAC
	Actin_R	CTGGTCTCAAGTCAGTGTACAGGT
ChIP	Region Far of Promoter_F	CAAGCATTAGGCAGTGTTGC
	Region Far of Promoter_R	GCTTGGGGCAACTTAGAAAA
	Region AP-1_F[17]	GCCTCCCAAGTTTGCTAATG
	Region AP-1_R[17]	TGTACTCTGGTATGGCACAAAGA
